# Integrating Nanostructured Artificial Receptors with Whispering Gallery Mode Optical Microresonators via Inorganic Molecular Imprinting Techniques

**DOI:** 10.3390/bios6020026

**Published:** 2016-06-15

**Authors:** G. Denise Hammond, Adam L. Vojta, Sheila A. Grant, Heather K. Hunt

**Affiliations:** Department of Bioengineering, University of Missouri, Columbia, MO 65211, USA; gdh52b@mail.missouri.edu (G.D.H.); alv5y2@mail.missouri.edu (A.L.V.); grantsa@missouri.edu (S.A.G.)

**Keywords:** molecularly imprinted polymers, Whispering Gallery Mode optical microresonators, optical biosensors, nanostructured thin films

## Abstract

The creation of label-free biosensors capable of accurately detecting trace contaminants, particularly small organic molecules, is of significant interest for applications in environmental monitoring. This is achieved by pairing a high-sensitivity signal transducer with a biorecognition element that imparts selectivity towards the compound of interest. However, many environmental pollutants do not have corresponding biorecognition elements. Fortunately, biomimetic chemistries, such as molecular imprinting, allow for the design of artificial receptors with very high selectivity for the target. Here, we perform a proof-of-concept study to show how artificial receptors may be created from inorganic silanes using the molecular imprinting technique and paired with high-sensitivity transducers without loss of device performance. Silica microsphere Whispering Gallery Mode optical microresonators are coated with a silica thin film templated by a small fluorescent dye, fluorescein isothiocyanate, which serves as our model target. Oxygen plasma degradation and solvent extraction of the template are compared. Extracted optical devices are interacted with the template molecule to confirm successful sorption of the template. Surface characterization is accomplished via fluorescence and optical microscopy, ellipsometry, optical profilometry, and contact angle measurements. The quality factors of the devices are measured to evaluate the impact of the coating on device sensitivity. The resulting devices show uniform surface coating with no microstructural damage with Q factors above 10^6^. This is the first report demonstrating the integration of these devices with molecular imprinting techniques, and could lead to new routes to biosensor creation for environmental monitoring.

## 1. Introduction

Environmental pollutants, and, in particular, emerging environmental pollutants, such as agricultural contaminants (pesticides, herbicides, *etc.*) and veterinary pharmaceutical by-products, represent one of the largest and most challenging classes of pollutant chemicals that can have a significant, detrimental impact on human health and the environment. For example, pesticides and herbicides are classified by Environmental Protection Agency as two of the most toxic classes of environmental pollutants [1,2,3]. One of the most dangerous effects of these pollutants is that they are capable of causing serious health problems, such as bone marrow disease, increased infertility, and increased instances of immunological and respiratory diseases, at relatively low prevalence [2,3,4,5]. Hence, it is essential to have an accurate method that is capable of trace detection of these pollutants in food and water systems. Currently, the primary method for detecting and identifying these pollutants is chromatography, including GC-MS and HPLC, which can accurately detect and identify these compounds from water samples extracted from the environment [6]. However, there are significant disadvantages to these methods that can interfere with adequate monitoring: for instance, the requirement for a library of data from high purity standards of all possible pollutants, the extensive sample preparation time needed, and the high equipment costs, in addition to the general lack of portability of these methods. Therefore, significant improvement is needed to address the ever-increasing complexity and pervasiveness of environmental pollutants in food and water systems.

Recently, biosensors, and in particular, optical biosensors, have been suggested as a possible alternative to chromatography-based techniques [7,8,9]. Optical biosensors have shown tremendous promise for numerous applications in medical diagnostics, and more recently, national security, and environmental monitoring applications [10,11,12]. In general, their utility arises from their non-destructive sample interrogation methods, their ability to perform extremely sensitive detection in liquid environments, and their high signal-to-noise ratios compared to other signal transduction methods, due to their relative immunity to the environment or system noise from electrical and mechanical sources [13]. Traditional optical sensors are typically labeled devices, which detect a label on the target of interest (such as fluorophores, enzymes, nanoparticles, *etc.*), rather than the target itself [14]. Label-free optical sensors can offer better device stability, quicker response, and potentially enhanced performance, particularly when tracking molecules whose behavior may change due to the presence of the label. Labels are typically used to increase the signal-to-noise ratio; label-free sensors have an inherently high signal-to-noise ratio, negating the need for a label. Many label-free optical sensors are refractometric sensors; that is, they function by detecting small changes in the effective refractive index of their optical field, which allows them to perform fast detection of minute changes in the refractive index of the optical field at the sensor surface/sample interface. These sensors, which include optical fiber sensors, Surface Plasmon Resonance (SPR) devices, and Whispering Gallery Mode (WGM) devices, have become increasingly popular due their speed, sensitivity, and label-free sensing properties [15,16]. Recently, a biosensing platform using an ultrahigh-Q microcavity has been reported for the detection of the Influenza A virus and polystyrene nanobeads. Although this method used a microtoroid platform, it can be applied to other type of WGM devices, such as silica microdisks and microspheres [17]. In this study, we focus specifically on silica microsphere WGM optical microresonators as the device of interest.

WGM optical microresonators are a type of circular resonant cavity device that confines light around their periphery via total internal reflection. WGM optical microresonators can be fabricated in a number of geometries, such as spheres, toroids, disks, and rings, both on-chip and free-standing [18]. In this study, we use silica microspheres of approximately 100 μm in diameter as the optical microresonator. Because of the low sorption of light by silica and their atomically smooth surface, these devices typically have very low loss, so that when light is coupled into these devices, it can circulate around these microspheres over 100,000 times [19,20]. While the light is reflected around the periphery of the device, it is not totally confined, but instead extends into the surrounding environment through the process of evanescence, allowing the field to interact with both the device surface and the surrounding environment [20]. Each time the optical field circulates around the device, it has an opportunity to interact with the environment, resulting in the amplification of the single-pass sensitivity that would be present if the device was linear. The photon lifetime in the resonator is measured by the Quality (Q) Factor of the device. A longer photon lifetime results in higher Q Factors. In sensing, the adsorption of an analyte onto the device’s surface causes the circulating optical field to undergo a change in its effective refractive index, thus inducing a rapid and measurable shift in the resonant frequency of the circulating field. This is the fundamental basis for the sensing capabilities of WGM optical microresonators, as well as their inherently high sensitivity [21].

When these devices are paired with an appropriate recognition element, they are also capable of high *selectivity* detection and target identification [21,22,23,24]. Typically, these recognition elements will target a compound of interest through biological molecular recognition processes, such as antibody-antigen binding, hormone-receptor binding, *etc.* Previously, a number of routes to adding selectivity to label-free optical biosensors, such as WGM optical microresonators, has been explored. These routes include physical adsorption, the generation of self-assembled monolayers, the use of covalent binding (or other grafting-type approaches, including the use of inorganic-organic coupling agents, such as silane molecules, polymer brushes, *etc.*), and the generation of sandwich-type attachment using the streptavidin- biotin complex [21,24]. These have resulted in surfaces modified to present a number of different receptors, including nanoparticles [25,26,27,28], polymers [19], and a host of biological substances, including proteins, antibodies, oligonucleotides, *etc.* [21,29,30,31,32]. These devices, when integrated with the appropriate recognition element, have performed ultra-low detection of trypsin, thrombin, DNA, and streptavidin, among many others [24,33]. Recently, another method that includes a WGM-nanoshell hybrid resonator has been used to detect MS2, which is the smallest RNA virus. Further, the detection of the thyroid cancer marker, Thyroglobulin, and bovine serum albumin has been demonstrated with WGM-h microcavities that were created by adding a single gold nanoshell to the equator of the WGM resonator [34].

However, many biologically relevant compounds, including many emerging environmental pollutants, do not have correspondingly specific biorecognition elements. Fortunately, biomimetic chemistries, such as molecular imprinting, allow for the design of nanostructured, artificial receptors, based on shape, size, and functional group selectivity, that have greater stability than most biological recognition elements and the potential for very high selectivity [35,36]. The molecular imprinting technique offers a promising alternative to the use of traditional biorecognition elements, and has been used extensively for the development of high selectivity optical biosensors [23,35,37]. In general, artificial receptors created via the molecular imprinting of a target molecule are stable at a wide range of temperatures and pH, are typically less costly to create than natural biorecognition elements and easier to process, can be produced for almost any compound, and are significantly more compatible with the typical inorganic surfaces that comprise most label-free optical sensor surfaces than traditional biorecognition elements [36].

The first attempt of using Molecularly Imprinted Polymers (MIPs) as a recognition element for biosensors was made in 1992 [36]. Since that time, MIPs have become an important class of synthetic, nanostructured material whose ability to selectively adsorb and otherwise interact with a variety of compounds has seen use not only in sensing applications but also in chemical and biological separations [38,39]. The term molecular imprinting refers to Fischer’s ‘lock and key concept’ [40], which explains the specific action of an enzyme with a single substrate, where the lock is the enzyme and key is the substrate. This theory helps to illustrate the specificity of enzymes towards a certain substrate. In addition, Pauling’s ‘production of antibodies *in vitro*’ [41] and Dickey’s ‘specific adsorbents’ [28] influenced the initial design of MIPs structures. However, “modern molecular imprinting was clearly established by Wulff and Mosbach, and their pioneering work has led to the current flourishing of molecular imprinting” [42]. Since then, the molecular imprinting technique has proven to be an effective technique for the creation of specific recognition sites in polymeric matrices.

The molecular imprinting technique is accomplished by polymerizing (typically organic) monomers in the presence of the target compound, which acts as a template around which the polymer network forms, and with which the network interacts. After polymerization, the template is removed from the polymer host network, resulting in a three-dimensional, nanostructured, porous polymeric network that theoretically is capable of reabsorbing the template molecule—and only the template molecule, due to the strong interactions between the template and host matrix. This process creates artificial recognition sites that have a high affinity for the template molecule [43].

MIPs created in this fashion can then be prepared in a variety of physical forms [44]. The traditional approach is to synthesize MIPs in bulk, then grind the resulting polymer and successively sieve the particles into the desired size ranges according to the specific application. Although this method is simple, the overall process is time-consuming and often produces non-uniform particles in terms or size or shape. Moreover, the grinding process might result in the destruction of some interaction sites, which reduces the material’s loading capacity [45]. Generally, macro, micro, and nano-sized interaction sites are formed from the monomers simultaneously as a result of this process. Therefore, it is hard to optimize the binding properties of bulk MIPs [46]. An alternative approach is the creation of MIPs in thin layers (such as conformal coatings or thin films). Although both methods provide an imprinting effect, and result in reasonable specificity, and selectivity, thin layer MIPs result in significantly better outcomes in all areas [47]. Recently, the literature has reported the creation of membranes, *in situ* prepared monoliths, surface imprinting, and monolayers through a thin layer approach [48]. Additionally, bulk MIPs are challenging to appropriately integrate with a high-sensitivity optical signal transducer, which is usually quite sensitive to the impact of surface treatments on its overall performance. Therefore, if MIPs are to be integrated with a high sensitivity, label-free optical signal transducer, such as a Whispering Gallery Mode optical microresonator, a thin layer MIPs procedure will be required to obtain a well-adhered, conformal coating that does not negatively impact the underlying device performance. Previously, there have been studies demonstrating the coating of single layer of molecular gain media on WGM optical resonators [49,50]. However, this study represents a coating that is nanostructured and theoretically able to act as an artificial receptor and therefore able to respond selectively to various target molecules.

However, there are some challenges that exist in terms of integrating surface coatings with micro/nanoscale, often fragile, optical devices. Surface coatings applied to WGM optical microresonators may decrease the sensitivity due to damage the coating process may cause to their glass structure. In general, these devices do not respond well to traditional wet chemistry techniques, which significantly limits the approaches that may be taken to create a conformal coating of a nanostructured, polymeric network that can act as a source of artificial receptors. Furthermore, they often require inorganic-organic linkers in order to be bound to the surface to ensure good adhesion between the surface and the recognition element or the coating. Hence, in this study, the possibility of creating of an artificial receptor via the molecular imprinting technique using *inorganic silane* polymer precursors, which can covalently bond to the underlying silica device surface, is examined. The expected (and achieved) result is the creation of nanostructured, inorganic polymer that is compatible with and capable of good adhesion to the underlying device.

The choice of a target molecule (and, therefore, MIPs templating agent) was based upon the need to easily confirm its presence or absence using well-accepted techniques, such as microscopy and spectroscopy, and upon the requirement that it be reasonably sized, such that it can act as a model target for common environmental pollutants. Therefore, fluorescein isothiocyanate (FITC) was chosen as the target molecule, as it satisfies the aforementioned conditions. In order to promote adhesion and conformal coating to form an inorganic polymer network around the template, silane precursors were used. To ensure high quality coatings, different types of coating methods were examined, including dip coating by hand and via automation. In addition, different types of extraction methods were studied to determine an appropriately effective route to template removal. Optical performance of the devices was measured in term of Q factors, both before and after coating and template removal, which allowed the quantification of the impact of the coatings on the devices. This study represents the first example of pairing silica microsphere WGM optical resonators with molecularly imprinted polymers, and, in particular, an inorganic polymeric network. Therefore, it should serve as an initial guide for designing appropriate solutions for the detection of emerging environmental pollutants using artificial receptors.

## 2. Materials and Methods

### 2.1. Preparation of Whispering Gallery Mode Optical Microresonators and Control Surfaces

**Figure 1:**
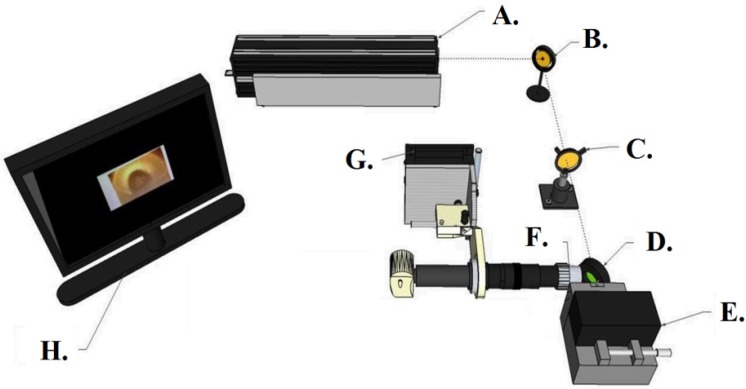
Schematic of the CO_2_ laser setup used to produce silica microspheres of approximately 100 μm diameters. **A**—CO_2_ Laser; **B**—Mirror; **C**—Focusing Lens; **D**—Beam Splitter; **E**—Sample Stage; **F**—Microscope; **G**—Microscope Alignment Block (2-axis Stage); **H**—Computer.

Microspheres were created by gravimetrically melting the tip of a stripped, single-mode optical fiber with a 25 W CO_2_ laser (Synrad, 48-2) operating at 7%–10% power. Microspheres should be above 40 μm in diameter to minimize diffraction losses; typically, they are between 100 and 200 μm in diameter. The primary advantage of using the CO_2_ laser for the fabrication of microspheres is that the resulting microsphere has an atomically smooth surface that is free of defects [51]. To achieve this, a small section of optical fiber (F-SC, Newport, CA, USA) is sliced to obtain a 4–7 cm length of fiber. On one end of the fiber, 1 cm of the protective coating is stripped off using a fiber stripper (No-Nik). The stripped portion is then cut down to approximately 2 mm with using a bare fiber cleaver. The prepared section of optical fiber is then cleaned with methanol using a Kimwipe to remove any loose protective coating. The optical fiber sample is then placed vertically into the path of the CO_2_ laser by attaching the fiber to a 3-axis stage. The fiber is then located in the cameras used to image the microsphere screen by moving the stage via the *Z*-axis motor controller (ThorLabs, Newton, NJ, USA). When the stripped end of the fiber appears in the center of the screen, the laser is fired. Approximately 3 s into the lasing process, the tip of the fiber starts forming a liquid droplet. When the ideal shape is obtained, and no air bubbles are present inside the droplet, lasing is stopped, and the droplet solidifies into a microsphere (Figure 1).

The fabricated microspheres are placed into Petri dishes that contain a glass slide prepared for microsphere storing. Double-sided scotch foam tape is cut and placed horizontally on one end of the normal-sized microscopic glass slide. The stems of the microspheres are then carefully placed onto the tape. Since the tape has a thickness about 1.5 mm, the stripped end/microsphere end of the optical fiber is lifted above the glass surface, preventing the microsphere from touching the container. This reduces the contamination or surface damage that might occur. The Petri dishes are then covered and placed in a humidity- and dust-controlled cabinet until further use [52].

Silica-on-silicon wafers served as controls to determine the quality of the resulting thin layer for techniques that do not work well with silica microspheres, such as ellipsometry. Prior to treatment, the silica surface was cleaned by submersion in a beaker containing C_3_H_6_O that was placed for 20 min in an ultrasonic bath (Elma, LC20H); this method of cleaning proved more effective than wiping the wafers with KimWipes using DDI H_2_O (Fisher Scientific, Pittsburgh, PA, USA) and acetone (Fisher Scientific, 99.5%). After this initial cleaning, the silica surface of both the wafers and the as-fabricated silica microspheres was terminated with hydroxyl groups to improve adhesion and binding to the MIPs thin layer using literature procedures using either an O_2_ plasma treatment (Plasma Etch, PE-50, 202 mTorr, 15 and 30 min) or a 70:30 H_2_SO_4_ (fuming, Sigma-Aldrich, St. Louis, MO, USA): H_2_O_2_ (30 wt %, Sigma-Aldrich) piranha etch for 45 min [53,54,55]. Immediately after the hydroxylation step, the samples were cleaned with DDI H_2_O (Fisher Scientific, f.w. 18.02) and air-dried at room temperature for 1–24 h to remove water from the sample surface. Following drying, each silica wafer or microsphere was coated according to Section 2.3.

### 2.2. Characterization of the Optical Devices

**Figure 2:**
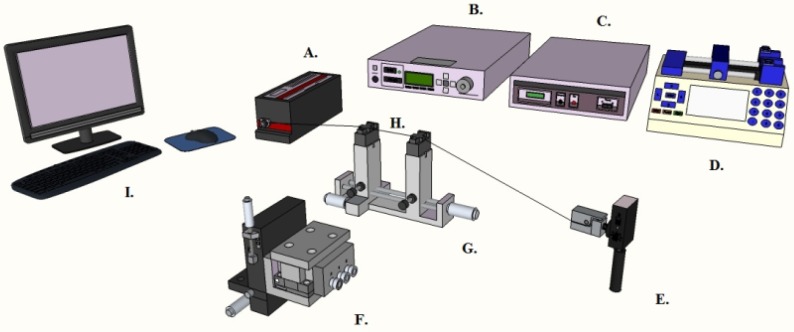
A model for the experimental setup used to determine the Q factors of the microspheres. **A**—Laser; **B**—Laser Controller; **C**—Stage Controller; **D**—Syringe Pump; **E**—Photo Detector; **F**—Nano-Positioning Stage; **G**—Taper Holder; **H**—Taper; **I**—Computer. Used with permission from Ref. [56].

Evaluation of the effect of the added artificial receptor coating on the device’s Q factor is essential to determining the utility of coating and the coated device. In order to achieve this, the Q factors of the devices are measured before coating, after coating, and after template removal. To do this, light is coupled into the devices from a narrow linewidth, CW tunable laser centered at 980 nm (New Focus) via a tapered optical fiber waveguide. During testing, the device is monitored simultaneously with side and top view cameras ((Moticam 1000, 1.3 M pixel) to enable for precise coupling between the microresonator and the optical fiber waveguide. The device itself is held within a microsphere holder attached to a 3-axis nanopositioning stage (Optosigma, Santa Ana, CA, USA) used to control the position of the microsphere relative to the tapered optical fiber waveguide (Figure 2). Resonance linewidth data that are obtained from this test are recorded using a digitizer/oscilloscope card that is integrated with the computer; this data are then input into a custom LabVIEW program, which simultaneously controls the laser system and the oscilloscope. The quality factor of the WGM microresonator under study is calculated from the recorded data by performing a Lorentzian fit to the resonance peak and abstracting the center wavelength and full-width at half-maximum from the peak. The Q factor is then calculated via the formula Q = λ/Δλ. The Q-factor data are obtained three times for each device: prior to coating, after coating, and post extraction.

### 2.3. Synthesis of MIPs

**Figure 3:**
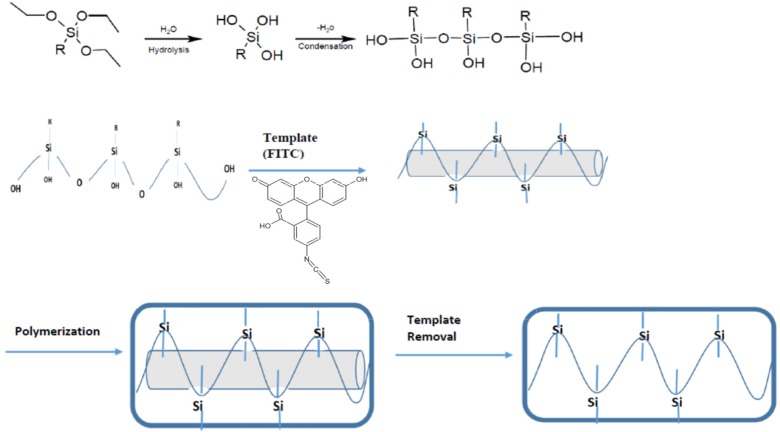
Molecularly Imprinting Technique using inorganic silanes as polymer precursors, and FITC as the template molecule. The silane precursors polymerize through hydrolysis and condensation reactions to form a three-dimensional, interconnected polymer network around the template.

The molecular imprinting technique results in the production of a nanostructured, polymeric network that is formed around the template molecule. This process is composed of three main steps. The first step includes choosing the monomer based on its potential interactions with a template molecule. Afterward, these monomers are polymerized in the presence of template molecules as the second step. The final step involves removal of the template molecule from this complex leaving the molecularly imprinted polymer with a pocket or binding site of the relative shape and size of that template molecule (Figure 3). Intermolecular interactions like hydrogen bonding, dipole–dipole bonding, and ionic interactions between the template molecule and functional groups present in the final polymer matrix drive the molecular recognition phenomena [45]. Thus, the resultant polymeric network “recognizes” and binds selectively to the template molecules. Most of the time, acrylate and methacrylic monomers are used for the preparation of molecularly imprinted polymers [36].

In this study, FITC is used to identify the key points of making the desired artificial receptor that is compatible with the material, geometry, and transduction mechanism of the proposed sensing system. The procedure for synthesizing a molecularly imprinted silica network is generally as follows: silica sol-gels are created by mixing 5 mL ethanol (95%), 440 μL methyltriemethoxysilane (Sigma-Aldrich, St. Louis, MO, USA, 99%), 14 μL aminopropyltriethoxysilane (Sigma-Aldrich, 98%), and 25 μL hydrochloric acid (HCL, 1 M, Sigma-Aldrich, 37%) in a glass beaker; the solution is stirred for 30 min at room temperature. 1.5 μL of a solution containing the template molecule is prepared by dissolving 1 mg FITC (Thermo Scientific) in 1 mL Dimethyl Sulfoxide (DMSO, Fisher Scientific, 99.9%), is then added to the solution, which is stirred for additional 1–2 min. The resulting sol is then coated onto either silica-on-silicon wafers or onto silica microspheres, via manual dip coating or automated dip coating (Thor Labs). The coating is followed by aging at room temperature, under darkened conditions (so as to reduce photobleaching) for a certain amount of days, ranging from 1 to 10, to yield a thin layer of polymer adhered to the surface of either the control wafers or the silica microspheres.

### 2.4. Optimization of Microspheres

During this study, the sol-gel formation parameters were varied to obtain high quality thin films on the surface, while minimizing the structural damage to the WGM resonators. The two main parameters were the coating techniques and the aging times, as shown in Table 1. Two different coating methods were tested: automated dip coating and manual dip coating. The coating time, 45–50 s, was kept the same for both coating methods. One dip was used for each method, as multiple dips resulted in coating thicknesses beyond 100 nm, which has the potential to exceed the thickness of the coating that enables the entire coating to be within the evanescent tail region of the circulating optical field of the device.

Another important parameter tested was the aging times of the silica sol-gels after they were coated onto the surface of interest. Fabricated optical devices were placed in Petri dishes as a group of 5. In the interest of observing the effect of aging time on the quality of the resulting coating on the devices, a range of aging times, from 1 day to 10 days, was tested. A total of 10 sets of silica microspheres was fabricated and then coated with molecularly imprinted silica sol-gels. Starting from day 1, each group was examined daily using Fluorescence Microscopy. Fluorescent images and measurements were obtained for each set of microspheres to determine the best aging time within the given range. Normalized Intensity values were obtained by the division of the average intensity value of the sphere to its area.

### 2.5. Extraction Procedure

As mentioned previously, the most important property of molecularly imprinted polymers is having specific binding sites for the target molecule. Typically, chemical extraction is the template removal method used with MIPs. Several chemical extraction parameters were tested and fluorescence intensity measurements were used to confirm extraction. Extraction Solution 1 was synthesized by combining the following chemicals: 4 mL Ethanol (95%), 1 mL Chloroform (Sigma-Aldrich, 99.5%), and 0.5 mL Acetic Acid (Sigma-Aldrich, 99.7%). An alternative solution (Solution 2) was prepared with 8 mL Ethanol (95%), 2 mL Acetonitrile (Sigma-Aldrich, 99.8%), and 1 mL Acetic Acid (Sigma-Aldrich, 99.7%). As shown in Table 2, two different extraction solutions, Solution 1 and Solution 2, as well as different stage conditions (stationary *versus* run on a tilt-tray) were analyzed.

A total of 4 Petri dishes were used (20 microspheres) in the extraction analysis. For each Petri dish, a different extraction solution and a stage condition was tested, resulting in a total of four extraction methods that were examined. Table 3 summarizes this information.

**Figure 4:**
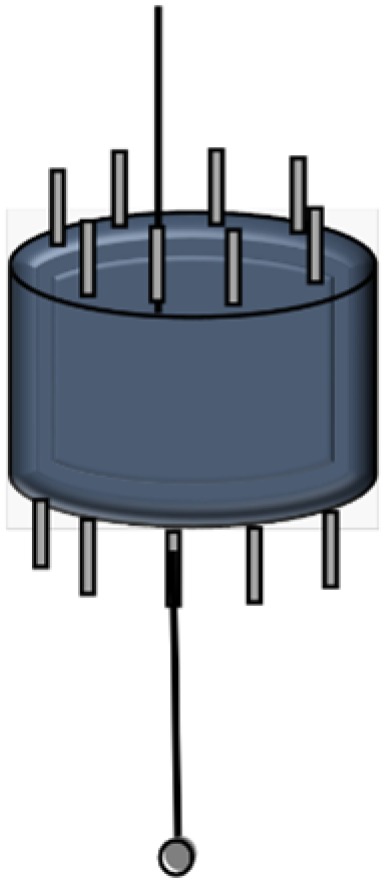
Schematic of a microsphere holder holding a single microsphere. This rubber stopper may then be placed inside a glass jar, suspending the microspheres in the solution without touching the side of the jars. This minimizes the possibility of damaging the microspheres due to handling. Up to eight microspheres may be suspended at once, although only one is shown here.

It was essential for the tips of the microspheres to be submersed in the extraction solution without touching the bottom of the container (to avoid microstructural damage). Thus, microsphere holders were used for stabilizing the positions of microspheres. A microsphere holder was made by inserting 8 very thin metal tubes (1–2 cm length) into a rubber stopper (Figure 4). After inserting microspheres through this tube, the rubber stopper was placed on top of the Erlenmeyer flask containing the extraction solution of interest; the flask was covered with aluminum foil.

### 2.6. Characterization of the Surface Coatings

Surface characterization of the coated wafers and silica microspheres was carried out using an Olympus IX70 inverted fluorescence microscope with an ORCA digital camera (Hamahatsu), before coating, after coating, post-template removal, and post-re-uptake of the template. Surface roughness of the thin layers on the wafers was evaluated using a Vecco NT 9109 optical profilometer using a PSI scan at 40× magnification and 0.5× field of view. To confirm the thickness of the coating on the wafers, ellipsometry was performed using a J. A. Woollam ellipsometer. A scan from 500 to 600 nm in intervals of 2 nm at angles of 65 and 75 degrees was performed; modeling via the Cauchy equations yielded an average thickness of the layer. Lastly, contact angle measurements were gathered from the wafers using a Ramé-Hart Model 200 Standard Contact Angle Goniometer for each the cleaned, hydroxylated, and coated wafers.

Further analysis of the microsphere coatings, and in particular, the loading capacity for FITC, was made with Ultraviolet-Visible Spectroscopy (Shimadzu, Columbia, MD, USA UV-2401). To be able to understand the coating chemistry, the initial sol-gel solution containing the template molecule FITC was analyzed before and after coating the microspheres to determine the concentration of FITC on the microspheres using the Beer-Lambert relation.

A set of 5 microspheres were fabricated and the silica sol-gel solution containing the template molecule was also synthesized. The calculated concentration for the MIPs solution containing the template was 15.2 M. In the first step, 1–1.5 mL of ethanol (Fisher Scientific, 95%) was added to the reference cuvette and the sample cuvette. This step was needed to create the baseline for the background, and ethanol was chosen as the baseline due to having a relatively high concentration in the solution mixture. After the initial spectra of the background solution was obtained, the second cuvette was emptied, cleaned, and refilled with 1–1.5 mL of the aforementioned MIPs solution. This step provided the spectroscopic data for the solution prior to coating. Next, the microspheres were coated using the MIPs solution, and after the coating was complete, MIPs solution that remained after the coating was analyzed in the second cuvette as before.

## 3. Results

In this study, we have used a series of microspheres to test different coating parameters and conditions. Table 4 below, shows a summary of samples utilized and the coating parameters to which they were subjected.

### 3.1. Characterization of the Uncoated Optical Devices

**Figure 5:**
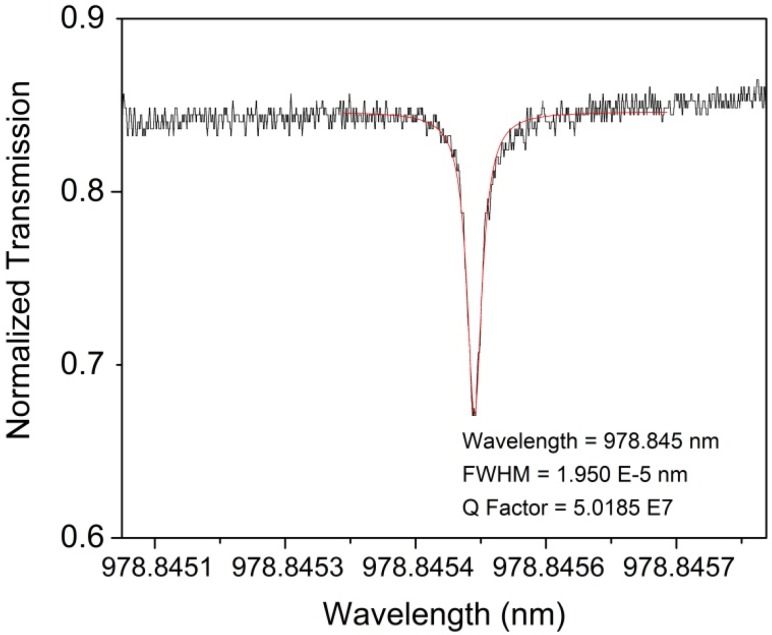
Q factor results of Dish 9 Sphere 1 (uncoated). Q Factors for the Dish 9 Spheres yielding consistent and high results above 10^6^ Q-factor measurements. The figure depicts a peak with the Lorentzian fit (**red** line) used to determine the center wavelength and full-width at half-maximum of the peak.

The microspheres in Dish 9 were tested to obtain typical Q-factor values of the bare spheres used in the project; these spheres were considered controls. All three microspheres had a Q factor value of 10^7^ magnitude, as expected, with an average Q factor of 1.241 × 10^7^. An example Q factor result for Dish 9, Sphere 1 is shown in Figure 5. It is important for optical devices to have high Q factor values before applying the MIPs coating so that, even if the coating decreases the Q factor, a good Q factor (typically defined as >10^6^, the lower limit for performing single molecule detection [57]) can still be obtained. Note that, with precise control of intrinsic device losses, such as material absorption (which is set when the wavelength and material are chosen), scattering (based on the quality of the surface and the presence of scattering sites), radiation (due to the curvature of the devices), *etc.*, intrinsic Q factors above 10^7^ can be attained [18]. In this study, the extrinsic Q factors devices were in the 10^7^ range, likely due to extrinsic losses from coupling.

### 3.2. Optimization of Microsphere Coating

Devices fabricated in this study were initially designed to target the FITC molecule. In order to create artificial receptors for this fluorescent dye, which exhibits a polyaromatic ring structure, we adapted the well-understood methods that are used to create MIPs for nitroaromatic explosives, which are of similar size and polyaromaticity. These methods were analyzed and transformed to allow for the production of inorganic silica sol-gel nanostructured coatings [58,59].

Here, we used two different silane monomers, and we evaluated the impacts of aging time and coating methods on the resulting quality of the coatings, as determined by Fluorescence Microscopy. Once the coating method was optimized, the aging time was analyzed. The aging window was expanded from 1 to 3 days to 1 to 10 days. The best aging time within this range was found according to the best normalized fluorescence intensity results (normalized as intensity per area under analysis, to allow for more accurate comparison between spheres and methods), which is discussed in Section 3.2.2. The template removal procedure was then investigated. Two different removal methods were tested: chemical extraction and oxygen plasma degradation. While chemical extraction is the typical template removal process for MIPs devices, it is a wet procedure, which can damage the microspheres. Template removal via oxygen plasma degradation, on the other hand, has been shown to work well with these devices, and does not require wet chemistry techniques [60]. Moreover, since oxygen plasma typically only degrades the organic molecule for the short time periods we evaluated, it is able to leave the silica coating intact, although it is likely that it will increase the formation of silanol groups on the surface. For the chemical extraction procedure, two different stage conditions and two different solutions were analyzed. Once the parameters were optimized, the best procedures for coating, aging, and extraction were applied to the control group of microspheres, which were tested at each coating step for their Q factor, as discussed previously.

#### 3.2.1. Coating Methods

We first evaluated the coating method (automated *versus* manual dip coating) to determine which resulted in higher-quality conformal coatings. Interestingly (and unexpectedly), switching from automated dip coating to manual dip coating led to a significant improvement in the coating results, as quantified by the optical and fluorescence microscopy images obtained. We surmise this was due to the rough/jerky start and stop of the coating equipment used, compared to the ability to finesse the coating by hand. Using manual dip coating, we were able to thoroughly control and consistently apply a conformal coating to the surfaces of the microspheres.

**Figure 6:**
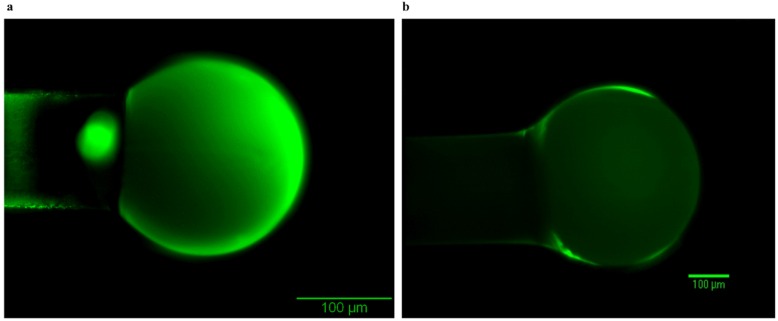
Dip coating methods applied to coat molecularly imprinted solution on the optical devices. (**a**) The result of the automated dip coating method, showing uneven saturation, and some microstructural damage (image from Dish 14 Sphere 30); (**b**) The result of the manual dip coating method shows uniform coating around the device (image taken from Dish 20 Sphere 4).

The results of this method are shown in Figure 6, which displays the uneven coating around the optical device (Figure 6a). On the other hand, manual dip coating results in a much better finish and even coating (Figure 6b). Images are taken with Olympus Widefield Fluorescence Microscopy. Therefore, the manual dip coating provides significantly better coating around the device than automated dip coating.

#### 3.2.2. Aging Times

**Figure 7:**
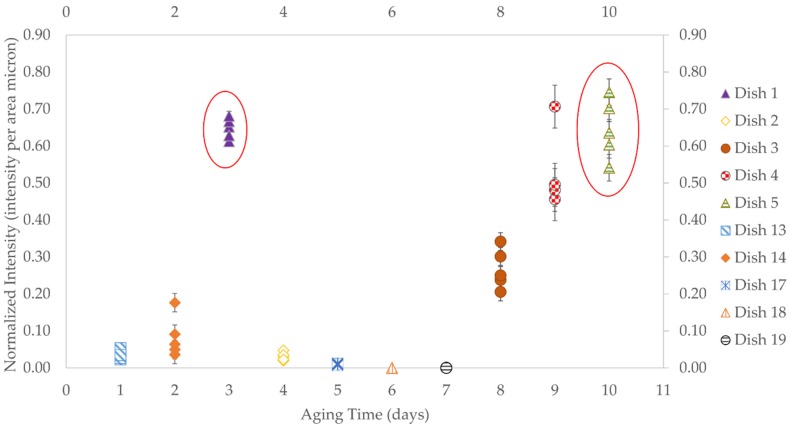
Normalized intensity *vs.* aging time. Each analyzed day includes a Petri dish that contains five coated microspheres. Day 3 and Day 10 shows the optimum results for MIPs coating procedure. Day 3 was chosen as it resulted in a more consistent normalized intensity and a shorter time-frame.

The result of this study showed that the optimum aging times for the microspheres was either 3 or 10 days (Figure 7). The fluorescence intensity measurements were the most important factors to determine the aging time. It was also observed that the fluorescence intensity generally decreased over the time until day 7 and then increased thereafter. Three days of aging was selected as the best parameter, due to its consistency and its shorter time-frame.

#### 3.2.3. Template Removal Procedure

**Figure 8:**
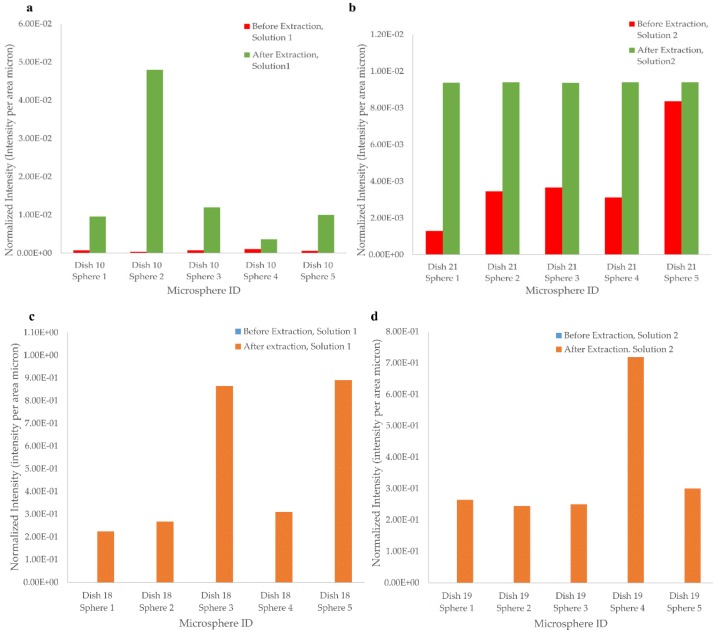
Results of the template removal via chemical extraction. Two different parameters, stage condition, and different solutions were tested. (**a**) and (**b**) shows the results for the tilt tray condition applied to Dish 10 and Dish 21; (**c**) and (**d**) shows the results for the stationary condition applied to Dish 18 and Dish 19. Chemical extraction results were not as expected; in fact, an increase in the fluorescence intensity measurements was observed. Therefore, several extraction parameters and different extraction methods were analyzed. Please note that, in figures (**c**) and (**d**), the pre-extraction data were so low in comparison, they do not show on the graphs.

It is expected that after the template removal, the fluorescence intensity exhibited by the coated microspheres will decrease. Early tests using Solutions 1 and 2 showed poor extraction results. Therefore, different parameters were tested to determine if the results could be improved. Two different extraction solutions, as well as two different stage conditions, were analyzed. Dish 10 was extracted with using Solution 1 and was placed on the tilt-tray (VWR incubating rocker), whereas, Dish 18, which was placed in the same extraction solution, was kept stationary. Moreover, Dish 21 was extracted using Solution 2 and was kept on the tilt tray during the extraction time. On the other hand, Dish 19 was kept stationary while being extracted with Solution 2 as well. Extraction time, 24 h, and reaction temperature, room temperature, were held constant for all conditions. The results, shown in Figure 8, suggest that there is an increase in the fluorescence intensity after the extraction procedure, which indicates that the extraction was not successful. This might have resulted from contamination that could have occurred during wet chemistry techniques (for instance, the molecules could have been extracted, and then re-absorbed onto the surface). Any extra residue that cannot be extracted via extraction solutions can cause increase in the fluorescent intensity. No significant difference was observed between Solution 1 and Solution 2 extractions.

The next step was to perform a template removal procedure using O_2_ plasma treatment. During this treatment, oxygen gas is introduced into the chamber. Like other forms of plasma, oxygen cleans organics and is capable of surface modification. Fortunately, the template molecule will be able to leave the MIPs complex intact, since it does not interact with inorganic surfaces as it does with organic materials.

**Figure 9:**
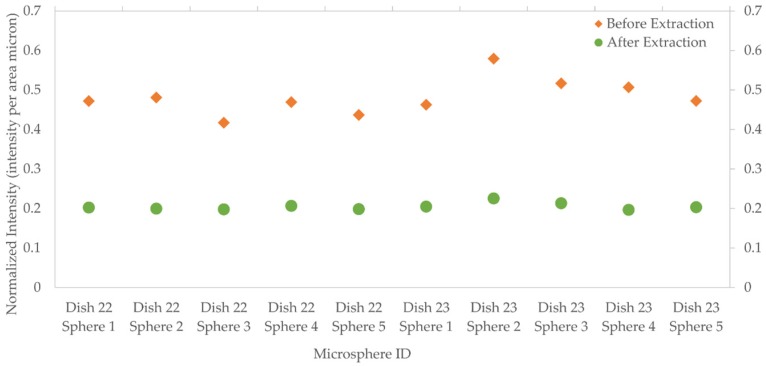
The result of normalized intensity values before and after the Oxygen plasma treatment taken from two Petri dishes, 22 and 23. Each Petri dish contains 5 spheres that are labeled with numbers for identification. A decrease in the normalized intensity is observed after the treatment suggesting that the template has been removed.

**Figure 10:**
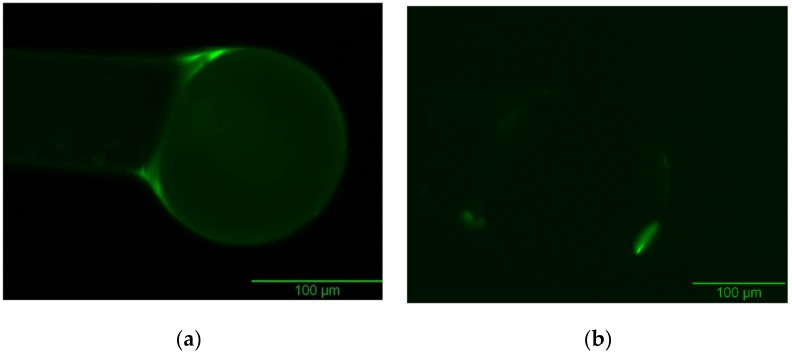
Images were taken from Dish 23 Sphere 3. Visible difference in the fluorescence intensity between two phases. (**a**) Pre-extraction; (**b**) Post-extraction. This particular image showed 59% loss in the intensity value after the extraction. The average intensity loss for this data set was calculated as 58%.

Oxygen plasma treatment, on the other hand, yielded better results, with significantly less risk of microstructural damage. Treatment parameters were varied until the best removal extent was measured; these parameters are presented in the Materials and Methods section. Figure 9 and Figure 10 show the results of this study. Although some FITC is still present in the template-removed coating, that is expected, as it is likely that the FITC closest to the sphere in the coating may be difficult to access via the oxygen plasma treatment, since the network of “pockets” for the template is not necessarily interconnected. Regardless, removal of the surface FITC will allow open pockets for template/target reabsorption.

#### 3.2.4. Q Factor Measurements

**Figure 11:**
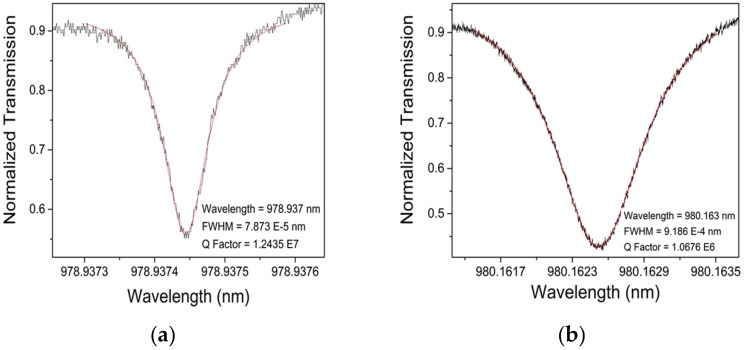
Q Factor graphs taken from a coated sphere before and after the extraction process. (**a**) depicts the fundamental peak of a coated sphere with a Lorentzian fit curve; the Q factor obtained was 1.243 × 10^6^; (**b**) depicts a similar peak on the same sphere following extraction via O_2_ plasma; its Q factor was 1.006 × 10^6^. Please note that the values given on the graph represent the direct output of the fitting and do not take into account the precision of the equipment. Transmission data (mW) were normalized based on the initial transmission of the laser.

**Figure 12:**
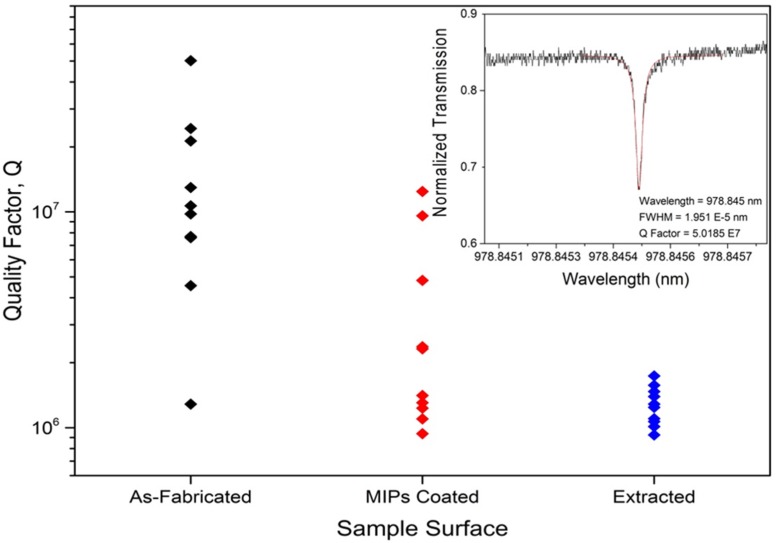
Q factors of all 10 spheres from Dish 22 and 23 taken as-fabricated, MIPs coated, and after the extraction process. The inset depicts the fundamental peak of an as-fabricated sphere with a Lorentzian fit curve; the Q factor was 5.0185 × 10^7^. As you can see the Q factor decreases at with each step in the procedure; and interestingly with a decrease in Q factor but less deviation after extraction.

Quality factors of the devices also represent significant evidence about the potential device performance. Previously, Q-factor values of uncoated spheres are presented which were recorded to be on average on the order of 10^7^. Figure 11 represents a sample Q-factor taken from coated microspheres both before and after template removal. Both graphs had a Q-factor that was higher than 10^6^ magnitude. These results show promising data in terms of using these devices in the further analysis. Figure 12 shows the results over all the spheres tested with the selected “best” parameters, and the impact of the coating on the Q factors.

### 3.3. UV-VIS Analysis

Through calculations of the absorption spectra, it was calculated that there was a 0.0008244-mole difference between pre and post-coating. This coating procedure involved 10 spheres in total, thus, the number of molecules ending up in the polymer network coating each sphere was approximately 4.965 × 10^19^ molecules of FITC, which represents the total FITC loading in the coating. On average, 58% template removal was obtained. One particular set, Dish 12, showed 73% template loss, resulting in uptake capacity of 3.62 × 10^19^ molecules of FITC per device. It is important to note that some of the MIPs coating also binds to a small portion of the microsphere stem, and thus the number of molecules coating each sphere itself would be some factor smaller. From fluorescence imaging, the normalized intensity could be found for all the parameters including the background intensity of the MIPs coating without the FITC template, the 3 and 10-day aging sets, post extraction, and finally reuptake of the template. Each set of measurements were averaged and their differences used to draw conclusions about the behavior and effect of the coating, extraction, and reuptake process (Table 5). This particular set, Dish 12 was extracted for 2 h using Oxygen plasma treatment.

These results show the normalized intensity for each step. Of note is the autofluorescence intensity from the MIPs solution without the FITC template and then the near total loss of fluorescence after O_2_ plasma extraction. The reuptake average is much lower than the initial coating measurements; however the loss of the autofluorescence intensity could be a large factor in this decrease.

### 3.4. Control Groups—Surface Quality of the Conformal Coatings

**Figure 13:**
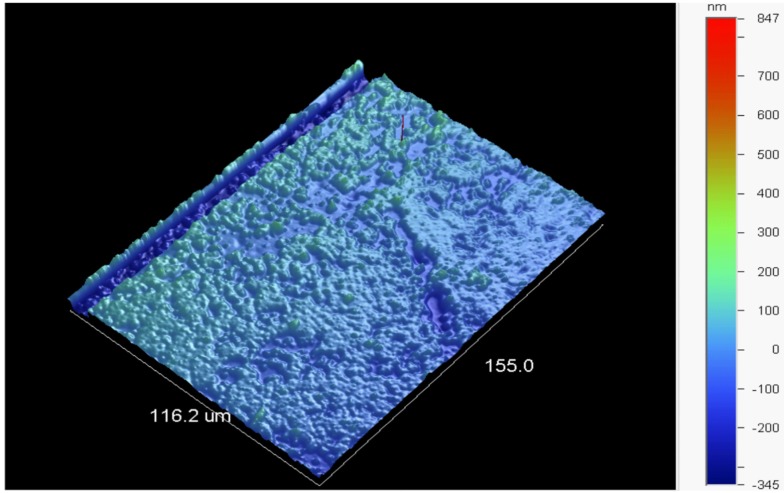
3D Image is taken using optical profilometry with 40.89 magnification and PSI measurement mode. Surface statistics for the sample shown: Ra = 50.14 nm, Rq = 75.29 nm, 1192.44 nm.

Surface roughness was calculated using a Vecco NT 9109 optical profilometer. Tests yielded consistent results across all parameters with an average surface roughness of 44 nm (Figure 13). Ellipsometry was performed to confirm the thickness of the coating on the wafers and an average of 70 nm was observed. Note that any increase in roughness of the microresonator’s surface will result in a direct decrease in the intrinsic quality factor that can be obtained from that microresonator. According to the work of Rahachou and Zozoulenko, quality factor reductions from theoretical values may arise due to geometrical imperfection, inhomogeneity in the microresonator in terms of material properties, *etc.* They focus specifically on the impact of surface roughness as an aspect of geometrical imperfection, showing that in the roughness range we report, high Q resonances will typically decrease by half an order of magnitude, which is similar to what is seen in this work [61]. Moreover, contact angle measurements were used and consistent measurements were obtained across all aging and hydroxylation parameters with an average contact angle of 73.16° ± 6.06°.

Piranha treatment has the possibility of being too harsh in terms of temperature and acid strength with respect to the structural and surface integrity of the devices. As an alternative, O_2_ plasma, which is typically used for cleaning residue from surfaces, is simple, minimizes device handling, and does not require post-treatment to remove excess water from the surface. On the other hand, O_2_ plasma treatment might not yield as high of a surface hydroxyl density as piranha etching, causing a lower adhesion of the coating to the surface. Therefore, it was important to identify which route is most compatible. In the end, O_2_ plasma treatment was chosen for such ease as no significant difference in measurements occurred between it and piranha etching.

## 4. Discussion and Conclusions

Whispering Gallery Mode optical devices fabricated in this study were initially designed to target the FITC molecule. In order to create artificial receptors for this fluorescent dye, a molecular imprinting technique was used. Integration of the artificial receptors with WGM resonators was the primary challenge; therefore, the primary goal of this study was to demonstrate the creation of a thin, uniform coating on the surface of the microresonators that was templated by and for FITC, while keeping underlying transducer undamaged. To achieve this goal, a thin film (<100 nm) is necessary to ensure that the coating, and any sorption interactions that could occur, remain within the evanescent tail of the device. The active region of the evanescent tail for devices that have a diameter of 50–100 μm is around 100 nm [62,63].

One of the challenges in this study was the selection of appropriate solvents in order to create the inorganic polymer network. However, by using prior literature results for both the creation of silica sol-gels and for the creation of organic MIPs for explosives detection, we were able to identify both good pre-cursors as well as solvents that interacted well and allowed the FITC molecule to still fluoresce. Once the solvents were selected as silica precursors, molecularly imprinted polymer synthesis studies were established. Artificial receptors with on-chip silica microsphere WGM resonators are created by gravimetrically melting the tip of an optical fiber with CO_2_ laser. Two of the most important key points during the microspheres production were having no air bubbles inside the device and obtaining a high Q factor. Several parameters were observed and tested during MIPs procedure in order to acquire the best quality coatings. The results showed that manually dip coating was the best selection in order to obtain a uniform coating around the sphere. The optimum aging time was determined as 3 days and 10 days; 3 days was selected due to its shorter time-frame. The template extraction method that gave the desired results was the oxygen plasma treatment, varying from 15 min to 2 h. Oxygen plasma treatment successfully removed a significant portion of the template molecule, which is confirmed by the difference between pre-extraction and post-extraction fluorescence intensity values. Furthermore, early extraction results suggested that the non-templated MIPs solution tends to auto-fluoresce. Therefore, it is important to take intensity readings without giving much time for the polymerization complex to auto-fluoresce. Otherwise, the extraction result is not verifiable since post-extraction intensity values might be higher than the pre-extraction values, which indicate that extraction might have been unsuccessful. Uptake of the template molecule after the extraction was confirmed via fluorescence intensity measurements. Finally, silica wafers were used as control groups to support the findings of the optimization studies. The results of the same procedures applied on silica wafers also proved that selected factors were the most appropriate.

The fundamental goal of this work is the creation of novel, highly sensitive and highly selective label-free optical sensor platforms that can be used to detect harmful environmental chemicals in trace quantities. The long-term goal of this work is to develop a generic, robust biosensor platform that can be arrayed to address multiple environmental pollutants, such as herbicides and pesticides, through minor adjustments to the artificial receptors.

## Figures and Tables

**Table 1:** Summary of optimization parameters tested in order to maximize the performance of the WGM optical resonators.

Optimization Parameters	Tested Methods	
Coating	Automated dip coating	Manual dip coating
Aging Times	1–3 days aging	1–10 days aging
Extraction	Chemical extraction	Oxygen Plasma extraction

**Table 2:** Summary of the type of extraction solutions used and the parameters tested for each solution.

Extraction Solution	Chemicals Used	Extraction Stage	Extraction Time
Solution 1	4 mL Ehanol, 1 mL Chloroform, 0.5 mL Acetic Acid	Tilt-tray, Stationary	24 h
Solution 2	8 mL Ethanol, 2 mL Acetonitrile, 1 mL Acetic Acid	Tilt-tray, Stationary	24 h

**Table 3:** Summary of extraction procedure. Dish 10, Dish 21, Dish 18 and Dish 19 were used for this procedure. Extraction time, 24 h, and extraction temperature, room temperature was kept constant for all conditions.

Petri Dish ID	Sphere	Stage Condition	Extraction Solution
Dish 10	1–5	Tilt-Tray	1
Dish 18	1–5	Stationary	2
Dish 19	1–5	Stationary	2
Dish 21	1–5	Tilt-Tray	1

**Table 4:** Summary of spheres used in this study and their identifications along with tested parameters. All the spheres in a Petri dish were treated exactly the same way as the other spheres in the particular Petri dish.

Microsphere ID		Coating Conditions		Extraction Conditions			
Petri Dish	Spheres	Coating Method	Aging Time	Extraction Method	Extraction Solution	Extraction Stage	Extraction Time
Dish 1	1–5	Manual	3 days	NA	NA	NA	NA
Dish 3	1–5	Manual	8 days	NA	NA	NA	NA
Dish 4	1–5	Manual	9 days	NA	NA	NA	NA
Dish 5	1–5	Manual	10 days	NA	NA	NA	NA
Dish 9	1–3	Uncoated	NA	NA	NA	NA	NA
Dish 10	1–5	Automated	3 days	Chemical	Solution 1	Tilt-Tray	24 h
Dish 12	1–3	Manual	10 days	O_2_ Plasma	NA	NA	2 h
Dish 13	1–5	Automated	1 days	NA	NA	NA	NA
Dish 14	1–5	Automated	1 days	NA	NA	NA	NA
Dish 17	1–5	Manual	5 days	NA	NA	NA	NA
Dish 18	1–5	Manual	6 days	Chemical	Solution 2	Stationary	24 h
Dish 19	1–5	Manual	7 days	Chemical	Solution 2	Stationary	24 h
Dish 20	1–5	Manual	3 days	NA	NA	NA	NA
Dish 21	1–5	Manual	3 days	Chemical	Solution 1	Tilt-tray	24 h
Dish 22	1–5	Manual	3 days	O_2_ Plasma	NA	NA	15 min
Dish 23	1–5	Manunal	3 days	O_2_ Plasma	NA	NA	30 min

Please note: NA: not applicable.

**Table 5:** Summary of different fluorescence parameters tested and their results.

Fluorescence Parameters	Normalized Intensity
Autofluorescence	0.193
3 Day Aging	0.648
10 Day Aging	0.645
Post Extraction	0.031
Reuptake	0.332

## Abbreviations

The following abbreviations are used in this manuscript:

MIP	Molecularly Imprinted Polymer
WGM	Whispering Gallery Mode
FITC	Fluorescein isothiocyanate
HCL	Hydrochloric Acid
DMSO	Dimethyl Sulfoxide
